# Studying upper-limb kinematics using inertial sensors: a cross-sectional study

**DOI:** 10.1186/s13104-015-1517-x

**Published:** 2015-10-03

**Authors:** Cristina Roldán-Jiménez, Antonio I. Cuesta-Vargas

**Affiliations:** Department of Physiotherapy, Faculty of Health Sciences, Instituto de Investigacion de Biomedicina de Malaga (IBIMA), Universidad de Malaga, Av/Arquitecto Peñalosa s/n (Teatinos Campus Expansion), 29009 Málaga, Spain; School of Clinical Science, Faculty of Health Science, Queensland University Technology, Brisbane, Australia

**Keywords:** Kinematics, Assessment, Inertial sensor, Shoulder, Upper limb

## Abstract

**Background:**

In recent years, there has been a great interest in analyzing upper-limb kinematics in order to investigate scapulohumeral rhythm, as its alteration has been associated with shoulder joint complex injuries. The use of inertial sensors is presented as a convenient and portable analysis method for studying kinematics in terms of angular mobility and linear acceleration. The aim of this study was to analyze upper-limbs kinematics in the three anatomical axes, obtained by inertial sensors.

**Results:**

Descriptive graphics of analytical tasks performed were obtained. The main difference in mobility between the scapula and humerus was found in pitch axis for abduction (${\bar{\text{X}}}$ = 107.6°, SD = 9.3°) and flexion (${\bar{\text{X}}}$ = 113.1°, SD = 9.3°).

**Conclusion:**

The use of inertial sensors for human kinematics analysis is favorable. Although this study identified movement patterns, and supports inertial sensors as a useful device to analyze upper-limb kinematics, further studies with subjects with shoulder pathology to establish differences in movement patterns and scapulohumeral rhythm between healthy and pathological shoulders should be carried out.

## Background

The shoulder joint complex consists of a set of five joints: glenohumeral, subdeltoid, scapulothoracic, acromioclavicular, and sternoclavicular, which makes it the most movable joint, i.e., in three anatomical planes and axes [1].

In 1934, Codman provided an overview of shoulder biomechanics in which these five joints make a constant and continuous movement, highlighting that, in the so-called scapulohumeral rhythm, movement of the scapula and humerus occur simultaneously, and in cases where this rhythm is disturbed, injuries in this joint complex may occur [2].

Scapulohumeral rhythm disturbance is still considered to play a role in shoulder injuries [3]. There are studies that corroborate that subjects suffering from shoulder injuries, such as impingement syndrome, have differences in scapular kinematics compared to healthy subjects, as abnormal scapular kinematics implies a reduction of subacromial space, producing a compression of the rotator cuff tendon [4, 5]. In this way, rotator cuff fatigue, supraspinatus deficiency, and anterior deltoid activation is associated with the superior migration of the humeral head relative to the glenoid fossa during arm elevation [6–8]. Also, the presence of rotator cuff tears has been associated with a disruption of the normal pattern of glenohumeral motion during arm elevation in the scapular plane [9]. More recently, a study found that deltoid activity decreases while trapezius activity increases during arm elevation, which can be interpreted as a bigger mobility in scapulothoracic joint to compensate painful glenohumeral joint [10].

This leads to the need to demonstrate this in the clinical field, and so, in recent years, there have been investigations concerning the assessment of scapulohumeral rhythm coordination by various devices.

One of the techniques employed to analyze the dynamics of the upper extremity is biplane fluoroscopy, which has been used to study the minimum distance between the acromion and humerus during clinical testing, and the relationship in the scapulohumeral rhythm in movements performed at different levels [11, 12]. Electromagnetic systems have been used when analyzing the kinematics of the shoulder in order to describe the normal movement of the shoulder girdle [13, 14], such as the one named Polhemus FasTrak, which has been used to compare pathological shoulders with the healthy contralateral shoulder in functional activities [15] and to describe the range of motion necessary in the upper extremity to perform the activities of daily living [16]. Furthermore, the relationship between the humerus and scapula and how they make its motion has been studied by many other techniques, such as optoelectronic systems [17], inclinometers [13], three-dimensional (3D) computerized tomography [18], and calibration anatomical systems techniques [19].

Although the 3D study on the position of the shoulder joint complex and the plane of rotation by using magnetic devices goes back decades [20], recently a new technology has been introduced that was borrowed from the aerospace industry, mechanical engineering, and robotics, and has proven to be a promising development in the sphere of motion analysis and an accurate and reliable method in human mobility studies. These are small electromechanical sensors that use technology from accelerometers, gyroscopes, and magnetometers, providing the potential required for dynamic 3D motion analysis [21].

Motivated by its small size and portability, these sensors could be an attractive option for human motion analysis, and literature reviews already exist around these sensors. There is evidence of operational feasibility of these units in various clinical applications [22], or reviews of the reliability and validity of these sensors [21], as well as a study in which the main advantages and disadvantages of a variety of motion analysis systems in which these inertial sensors were included have been discussed [23].

Very recently, a novel method was presented that automatically identifies inertial sensors in human body segments during walking, including the upper extremities, and is able to be placed in arbitrary anatomical areas, which makes them more easy to use in biomedical applications [24]. Furthermore, a strong level of evidence has been found for the validity and intra-rater reliability in digitizing palpation of bony landmarks to define anatomical axes of joint kinematics of joint segments, among which are the upper limbs [25].

In addition to the angular mobility, there is both scientific and clinical interest in other kinematic aspects, such as speed or angular acceleration, which can offer new and more information [21]. A method of tri-axial accelerometer analysis has been employed including an approach of assessing the distribution of time spent in the functional use of extremities [26]. The reliability of inertial sensors for these properties has been studied in anatomical regions such as the lumbar spine, offering favorable results [27].

Because of the wide applicability in clinical sciences, there have been kinematics studies based on inertial sensors measure in the arm. Several protocols have already been developed for analyzing the scapulothoracic, humerothoracic and elbow joints [28], scapula [29] and scapulohumeral rhythm [30]. Also, a standardized protocol was proposed for measuring upper-limb movements [31]. Very recently, reliability and precision of scapula kinematic through inertial and magnetic measurement system (IMMS) has been studied in healthy subjects [32]. From a more functional point of view, an inertial-sensor-based motion detector for estimating mobility has been developed in upper-limbs daily activities such printing patterns in a paper or drinking [33]. Focusing on stroke patients and their rehabilitation, inertial sensors have been included in a motion tracking device [34] as well as part of a hybrid tracking system integrating additionally vision for arm motion [35].

Despite executed protocols featuring in the arm and shoulder girdle, it is interesting to strengthen upper limbs kinematic values in terms of mobility and acceleration during upper-limbs motion in healthy subjects, taken into account tridimensionality characterizing human motion. The purpose of this study was to analyze upper-limb angular mobility and linear acceleration in the three anatomical axes using four inertial sensors placed in humerus, scapula, sternum and forearm in healthy subjects during flexion and abduction analytical tasks.

## Methods

### Subjects

This cross-sectional study recruited healthy young adult subjects who provided inclusion and exclusion criteria, and that were interested in taking part of the project. Students from the Faculty of Health Sciences (University of Málaga) were chosen.

Inclusion criteria were: aged between 18 and 35 years old; body mass index (BMI) between 18.5 and 28; and right-handed. Subjects were excluded if they refused to participate in the study or they had consumed analgesics or non-steroidal anti-inflammatory drug (NSAIDs). Also, subjects with shoulder pathology were excluded. Informed consent was needed.

In total, a group of 11 subjects (8 men, 3 women) were included. Written informed consent was obtained from each individual. The study was approved by the ethics committee of the University of Malaga, Spain.

### Apparatus

Descriptive and anthropometric independent variables related to age, gender, weight, size, and BMI were included. Six physical properties were included corresponding to three dependent variables for each of three special axes: mobility angle (°) and linear acceleration (m/s^2^) along X, Y and Z axes.

**Fig. 1:**
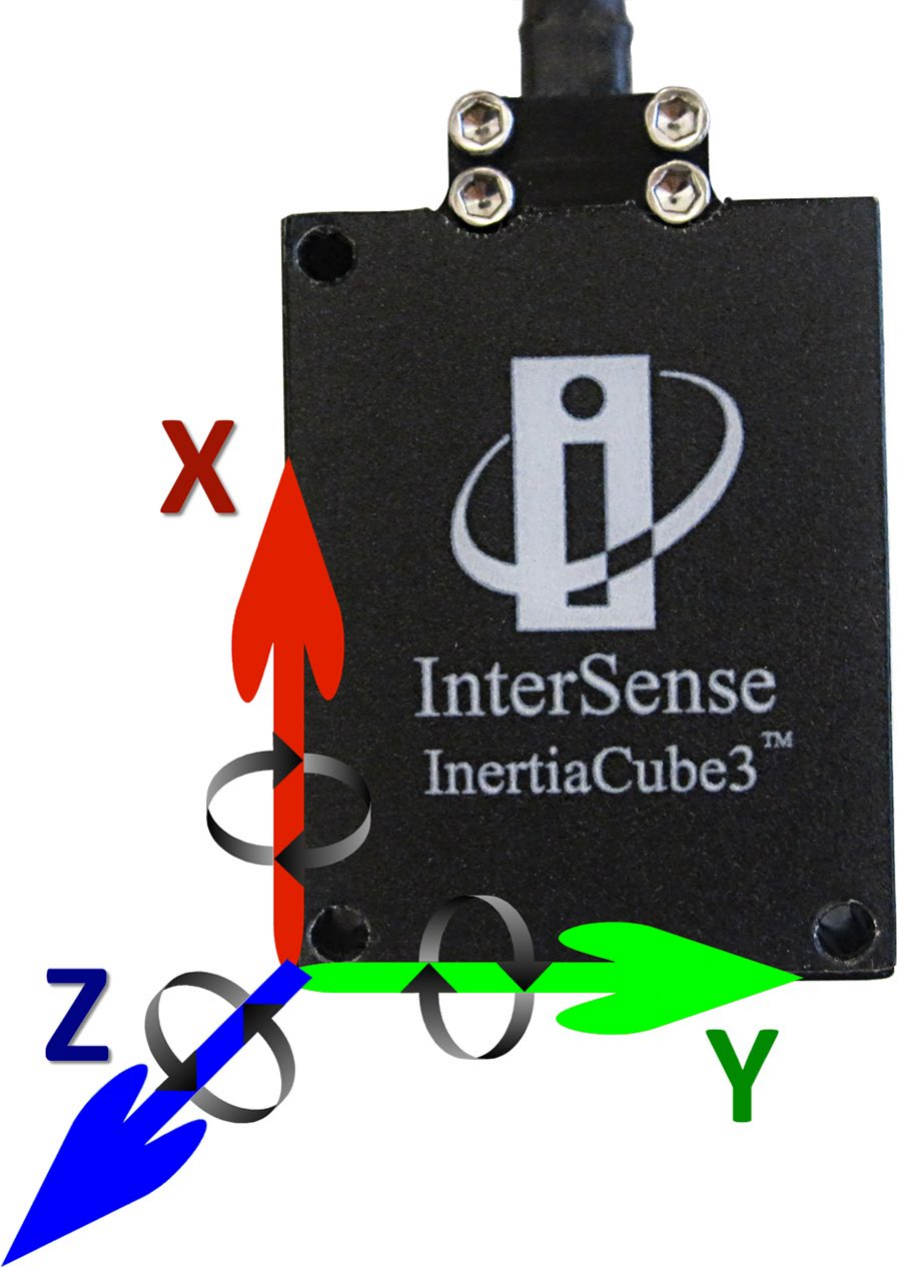
Representation of the 3 degrees of freedom in InertiaCube3™ sensor

These physical properties were obtained through the inertial measurement sensors with four inertial sensors (InertiaCube3™ Intersense Inc., Billerica, MA, USA) whose dimensions are 26.2 mm × 39.2 mm × 14.8 mm and weight is 17 g (Fig. 1). Each sensor contains an inertial 3-degree of freedom (DOF) orientation tracking system: X, Y, and Z, with an accuracy of 1°, 0.25°, and 25° respectively, an angular range of 360°, able to detect an angular rate between 0° and 1200° per second, with a sampling frequency of 1000 Hz.

Previous to their placement, sensors were reset at 0 using the Intersense Server Software. For this, they were placed on a horizontal flat surface and positioned vertically or horizontally according to their placement in the anatomical parts, which are described below.

**Fig. 2:**
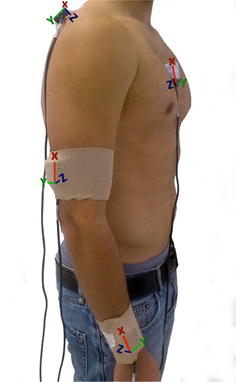
Placing 4 InertiaCube3™ sensors on the right hemi-body of a subject

Inertial sensors were placed on the right half of the body of each subject located in the middle third of the humerus slightly posterior, in the middle third of the upper spine of the scapula, in the flat part of the sternum, and the distal surface of the ulna and radius [28]. These surfaces were cleaned with alcohol in order to have each sensor adhere to the skin. To ensure fixation of the sensor to the subject’s skin and prevent slippage, a double-sided adhesive tape was used, as well as an 8 cm wide elastic cohesive (Rapidex^®^) to fixed cylindrical body segments (upper and lower arm), and an adhesive bandage 5 cm wide (Strappal^®^) in flatter areas of the body (scapula and sternum) (Fig. 2).

Because of their positioning, the axes in each of the sensors correspond to different planes of anatomical movement. Humerus kinematics was represented by sensor placed in humerus surface, and next motion terms were expressed: flexo-extension (FL-EX) along X axis, understood as humeral flexo-extension; axial rotation (IN-EX) along Y axis, understood as humeral internal and external rotation; and ab-adduction (AB-AD) along Z axis, understood as humeral abduction and adduction.

Forearm kinematics was represented by sensor placed in ulna and radius surface, expressing: flexion–extension (elFL–EX) along X axis, understood as elbow flexion; prono-supination (PR-SU) along Y axis, understood as elbow prono-supination; and carrying angle along X angle, understood as the relative orientation of the axes of the hinges.

Scapula kinematics was represented by sensor in placed in scapula surface, collecting next related axes kinematics: anterior–posterior tiling (AN–PO) along X axis, protraction–retraction (PR–RE) along Y axis, medio-lateral rotation (ME-LA) along Z axis.

**Fig. 3:**
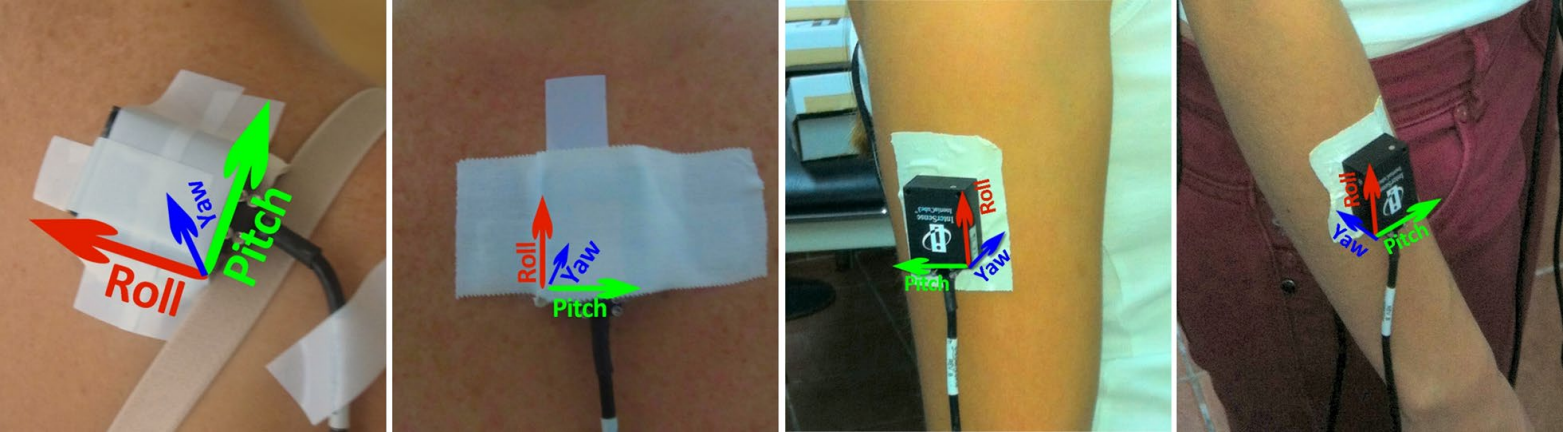
Representation of yaw, pitch and roll axes, in the four anatomical areas where inertial sensors are placed

Thorax kinematics was represented by sensor in placed in sternum, expressing: lateral rotation along X axis, understood as trunk lateralization; axial rotation along Y axis, understood as trunk rotation; and flexoextension along Z axis, understood as trunk flexo-extension (Fig. 3; Table 1).

**Table 1:** Equivalence of X, Y and Z axes with the movement that they represent

Surface placement:	Humerus	Ulna and radius	Scapula	Sternum
Anatomical segment represented:	Humerus	Forearm	Scapula	Thorax
Axis				
X	IN-EX	PR-SU	AN–PO	Axial rotation
Y	AB-AD	ElFL–EX	PR–RE	Flexion and extension
Z	FL-EX	Carrying angle	ME-LA	Lateral rotation

### Procedure

Activity values were recorded by kinematic Intersense Server Software, which were subsequently passed to a database of Microsoft^®^ Excel 2007. The wiring was placed so that it would not inconvenience the participant because this would impair task performances.

After study participant recruitment, they were asked to attend the study in the Human Movement Laboratory, Faculty of Health Sciences University of Málaga. Tasks were explained concisely and clearly so that the participant understood the action to perform. The beginning and the end were decided by a verbal order by the researcher. Participants were placed standing, starting from neutral position, performing the following analytical tasks:

1. 180° right shoulder abduction, with the elbow extended, wrist in neutral position and the palmar area of the hand toward the midline at the beginning and end of the movement (eight repetitions).
2. After a break of about 3 min, a further eight repetitions of the same task were performed.
3. 180° right shoulder flexion, with the elbow extended, wrist in neutral position and the palmar area of the hand toward the midline at the beginning and end of the movement (eight repetitions).
4. After a break of about 3 min, a further eight repetitions of the same task were performed.

### Data analysis

SPSS v15.0 was used for all statistical computations. Descriptive statistics (mean, standard deviation, minimum and maximum) were calculated for age, height, weight, BMI, angular mobility and linear acceleration. Standard procedures were used to calculate means and SDs. The Kolmogorov–Smirnov test showed a normal distribution of the data (P > 0.05). For all statistical comparisons, the α level was set at 0.05.

The highest point reached by the sensor placed in the humerus in the second repetition of the second series for both analytical task was used as the cut-off time, to which was subtracted the corresponding degrees obtained in the lowest point reached by each sensor in order to calculate angular mobility, expressed in degrees as range of movement or sensor displacement.

For calculating acceleration, expressed as °/s^2^, maximum peaks obtained in the second repetition of the second series for both analytical task, were subtracted the minimum peak obtained by each sensor.

When angular mobility of each sensor was obtained, joint angular mobility was calculated as the difference between two sensors. For this purpose, the range of movement obtained by the sensor placed on the body segment of interest on its main motion axis was rested to the range of motion obtained by a different sensor, as follows: glenohumeral joint—represents humerus relative to scapula, scapulothoracic joint—represents scapula kinematics relative to sternum; elbow joints—represents forearm kinematics relative to the humerus. Joint angular mobility was also calculated considering the resultant vector (Rv) of the three axes of movement, being understood as ${\text{Rv}} = \sqrt {x^{2} + y^{2} + } z^{2}$.

## Results

A total of 11 subjects (8 men, 3 woman) were measured, whose mean of age was 24.7 years (SD = 4.2 years) and their average BMI was 22.6 kg/m^2^ (SD = 2.2 kg/m^2^, Table 2).

**Table 2:** Values of anthropometric and descriptive variables

	Minimum	Maximum	Mean	Standard deviation
Age (years)	20.0	34.0	24.7	4.2
Size (cm)	156.0	184.0	172.1	9.1
Weight (kg)	48.0	87.0	67.5	11.7
BMI (kg/m^2^)	19.7	27.4	22.6	2.2

*BMI* body mass index

**Fig. 4:**
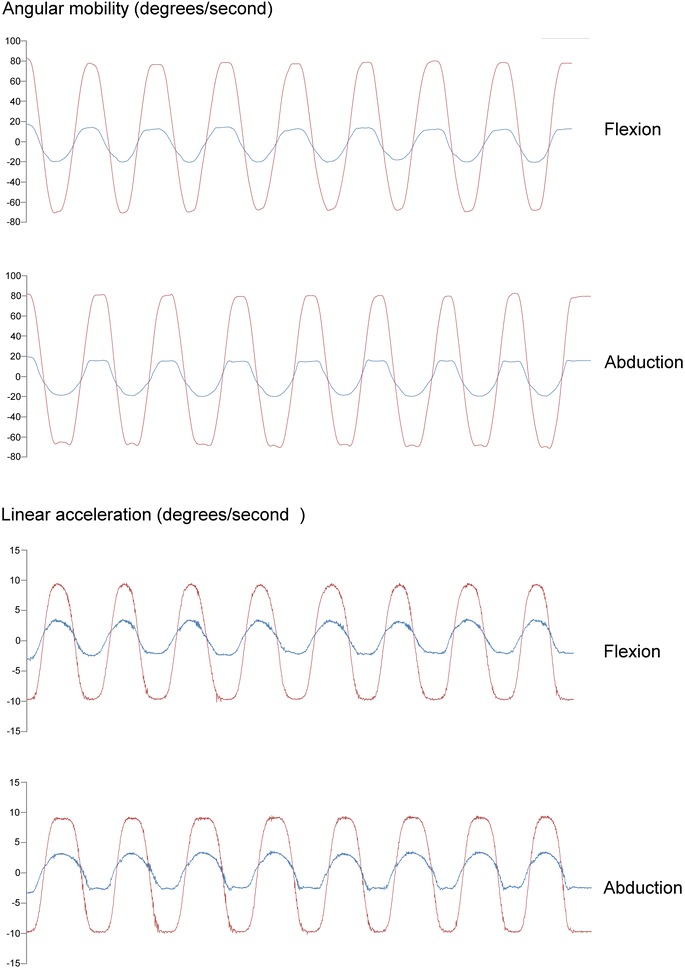
Kinematic pattern. Four examples of kinematic patterns through repetitions were showed for angular mobility in humerus AB-AD and scapula PR–RE in both analytical tasks. Humerus *red line*, Scapula *blue line*

Analyzing six physical properties that corresponded to three continuous quantitative variables (angular mobility, and linear acceleration) allowed us to obtain descriptive graphics of analytical tasks performed by each participant (Fig. 4).

Means and standard deviations of maximum acceleration peaks and difference angular mobility were calculated. Those variables corresponding to glenohumeral, scapulothoracic and elbow joints are expressed in Table 3 for abduction–abduction movement and Table 4 for flexo-extension task. In terms of glenohumeral joint, the bigger range of mobility was found in AB-AD for both analytical tasks, and a higher rotation value was found for in abduction. Regards to scapulothoracic joint, a bigger scapula displacement in all its component was found in flexion movement. Respect to elbow joint, results were similar for both movements, but was found a higher PR-SU in flexion, and higher FL-EX in abduction.

**Table 3:** Mean (SD) degrees of angular mobility from glenohumeral, scapulothoracic and elbow joints recorded in abduction task

Axis	Glenohumeral joint	Scapulothoracic joint	Elbow joint
Z	94.8 (36.7)	−5.9 (9.5)	29.3 (39.9)
	FL-EX	ME-LA	Carrying angle
Y	107.6 (9.3)	36.6 (10.2)	1.6 (5.9)
	AB-AD	PR–RE	elFL–EX
X	70.2 (31.1)	−5.5 (12.3)	16.9 (35.3)
	IN-EX	AN–PO	PR-SU
Rv	168.0 (36.8)	40.9 (7.7)	54.5 (29.7)

**Table 4:** Mean (SD) degrees of angular mobility from glenohumeral, scapulothoracic and elbow joints recorded in flexion task

Axis	Glenohumeral joint	Scapulothoracic joint	Elbow joint
Z	67 (36.7)	−7.7 (48.6)	24.8 (46)
	FL-EX	ME-LA	Carrying angle
Y	113.1 (9.3)	37.8 (6.3)	1.4 (11.9)
	AB-AD	PR-SE	elFL–EX
X	64.6 (31.1)	4.2 (16.9)	20.1 (32)
	IN-EX	AN–PO	PR-SU
Rv	154.9 (42.2)	53.5 (34.6)	56.9 (29.2)

Taking into account Rv, wider range of mobility was found in glenohumeral during abduction task, while this range was bigger for scapulothoracic and elbow joints during flexion.

Focusing on degrees provided by humerus, highest values were found in FL-EX for both task. Even in abduction task, FL-EX mean value was bigger than AB-AD, although AB-AD component was found bigger in abduction task than in flexion. Rotation was found similar for both task (see Table 5 for more details).

**Table 5:** Humerus angular mobility (°)

Axis	Analytical task	Minimum	Maximum	Mean	Standard deviation
AB-AD	Abduction	47.8	156.0	108.0	37.9
FL-EX	Abduction	133.3	167.9	151.7	9.7
IN-EX	Abduction	37.4	142.8	85.6	37.0
AB-AD	Flexion	23.4	168.4	87.8	46.1
FL-EX	Flexion	136.1	169.3	157.2	12.3
IN-EX	Flexion	15.8	145.3	85.4	48.4

With regard to acceleration, the highest average peak mean value was shown in the forearm in FL-EX motion: 20.1°/s^2^ for abduction and 19.8°/s^2^ for flexion followed by the humerus, scapula, and sternum (Table 6). Examples of kinematic patterns across repetitions traces during are showed in the Fig. 4.

**Table 6:** Acceleration (°/s^2^) in different motions during abduction and flexion tasks

Analytical tasks	Humerus				Scapula				Forearm				Sternum			
	Motion	Mean (SD)	Min	Max	Motion	Mean (SD)	Min	Max	Motion	Mean (SD)	Min	Max	Motion	Mean (SD)	Min	Max
Abduction	IN-EX	8.6 (1.7)	5.8	11.4	AN–PO	3.7 (1.6)	1.5	6.4	PR-SU	12.6 (2.7)	9.8	17.9	Axial rotation	2.0 (0.6)	1.4	3.5
Flexion	IN-EX	8.7 (2.7)	3.63	12.2	AN–PO	3.1 (1.4)	0.9	5.3	PR-SU	11.3 (2.5)	6.1	14.3		1.9 (0.4)	0.9	2.5
Abduction	AB-AD	19.4 (0.8)	18.11	21.19	PR–RE	7.8 (1.5)	6.0	11.2	elFL–EX	19.8 (0.6)	18.5	20.8	Flex and ext	1.8 (5.6)	0.5	19.7
Flexion	AB-AD	18.5 (0.8)	18.2	20.51	PR–RE	7.9 (1.3)	5.7	10.1	elFL–EX	20.1 (1.0)	18.6	22.0		1.2 (0.5)	0.6	2.4
Abduction	FL-EX	9.19 (1.6)	5.98	11.06	ME-LA	2.9 (1.1)	1.52	5.0	Carrying	9.7 (2.9)	6.3	16.3	Lateral rotation	1.7 (2.7)	0.4	1.7
Flexion	FL-EX	7.0 (1.8)	3.5	10.2	ME-LA	3.7 (1.3)	1.6	6.0	angle	6.4 (2.0)	3.0	8.8		1.3 (0.5)	0.5	2.1

Relationship between angular mobility and linear acceleration was calculated for both tasks in each anatomical axes. Strong correlation was found in Y axis for all sensors, as well as in X axis in humerus, corresponding to IN-EX movement, and in Z axis in forearm for flexion, corresponding to the carrying angle. More details are showed in Table 7.

**Table 7:** Pearson correlation (p value) between angular mobility and linear acceleration

Axis	Task	Humerus	Scapula	Forearm	Sternum
X	Abduction	0.72* (0.012)	0.33 (0.310)	0.52 (0.095)	0.84 (0.001)
	Flexion	0.72* (0.012)	0.43 (0.172)	0.23 (0.494)	0.41 (0.203)
Y	Abduction	0.91** (0.000)	0.85** (0.001)	0.74** (0.009)	0.17 (0.609)
	Flexion	0.92** (0.000)	0.75** (0.007)	0.64* (0.031)	0.82** (0.002)
Z	Abduction	0.31 (0.340)	0.00 (0.993)	0.51 (0.105)	0.06 (0.851)
	Flexion	0.23 (0.493)	0.52 (0.096)	0.61* (0.046)	0.13 (0.685)

## Discussion

The present study has described and examined shoulder angular mobility and linear acceleration while performing abduction and flexion movements through four inertial sensors placed in humerus, scapula, forearm and sternum in healthy subjects.

The results obtained in this study encourage the use of inertial sensors as a device for measuring upper limbs kinematics including angular mobility and linear acceleration in three anatomical axes, providing a tridimenstional view of humerus, scapula, forearm and trunk and how they contribute to shoulder joint complex kinematics.

With regards to humerus mobility, 151.7° in abduction and 157.2° in flexion was obtained by sensor placed in humerus. Nonetheless, a previous study focusing on humerthoracic joint, that is, humerus movement relative to the thorax, found 180° for both task in healthy subject [36]. Those differences are interesting because this study found lower values without removing thorax compensation. On the one hand, related to humerus motion, it is noteworthy that was found a bigger flexion component than abduction during abduction task, which also happens when analyzing glenohumeral joint. On the other hand, abduction component showed higher values in abduction task than flexion task, as might be expected.

There are several studies which have study scapula motion relative to the trunk. Through inertial sensors, a study using the same protocol, found 9.1° in AN–PO, 18.5° in PR–RE and 27.7° in ME-LA during shoulder flexion [28]. A more recent study based on mentioned protocol, found as average 9°–15° in PR–RE and 33°–36° in ME-LA for abduction and flexion respectively [31]. Using Polhemus Fastrak device, values were found to be 9.5° in AN–PO, 29.64° in ME-LA and 4.0° in PR–RE for abduction, and 5.0° in AN–PO, 27.06° in ME-LA and 16.8° in PR–RE for flexion task [37]. Focusing on scapular medio-lateral component, results provided by Cutti et al. [28] concur with those provided by Parel et al [29], a previous study that showed a 26.1° during flexion and 23.2° during abduction by using a scapular tracker from an optoelectronic system. Those results were similar when comparing with spinal tracker from a magnetic system [29]. However, subjects from this study showed negative ME-LA values during both task, suggesting great trunk compensation. Conversely, taking into account Rv, 40.9° and 52.5° were found for abduction and flexion respectively. The fact of obtaining different results depending on analyzing one plane or tridimensional movements intensifies the importance of taking into account the 3D component of anatomical movement, whose analysis is allowed through inertial sensors. Focusing on tridimensional motion, a wider range was found in glenohumeral joint during abduction, while scapulothoracic and elbow showed a wider range during flexion, supporting different tridimensional joint behaviors when performing motion in different planes. Also, a bigger rotational component was found in glenohumeral joint during abduction, while results were similar when focusing only in degrees provided by humeral sensors. Related to angular mobility, it should also be considered that high standard deviations were found in glenohumeral, scapulothoracic and elbow joints (Tables 3, 4). This may be due to the presence of peculiar cases dispersing data sample.

Concerning acceleration, higher average peak values were found in forearm, followed by humerus, scapula and sternum, respectively. Strong correlation was found between mobility and acceleration in humeral rotation and abduction component, forearm flexo-extension and scapula retraction and protraction in both tasks. Correlation was also strong for thorax in flexion–extension component during flexion task.

This study extends knowledge on the study on shoulder kinematics, offering mean values of mobility in different body parts and joints in composing shoulder complex, as well as boundaries of acceleration values in healthy subjects. Also, values provided correspond to each axes of motion, highlighting tridimenstional property of human movement. Previous studies have analyzed upper-limb properties through inertial measurement. Some of them studied a pair of corporal segments like humerus and sternum [36] or humerus and forearm [38]. However, this study used four inertial sensors, which provides more information about shoulder complex kinematics, being broadly in line with studies focusing on humerus, scapula, forearm and sternum [28, 30]. This also provides information from scapulothoracic and glenohumeral joint, whose muscular activity has been recently studied in both symptomatic asymptomatic subjects [10]. Even so, the contribution of sternoclavicular and acromioclavicular joints, whose importance has been previously claimed [39] was no estimated.

The main weakness of the study is that it is a cross-sectional study, and therefore, cause and effect relationships in kinematic patterns cannot be established. Furthermore, this study focused on analytical tasks, but did not analyse any functional task common in other studies, like hair combing (hand–head) or back washing (hand–back). On the one hand, having a sample with a larger number of participants and in which there are also subjects presenting shoulder pathology, we hope to compare our results with those that indicate that there is a difference in the shoulder mobility between healthy subjects and those with shoulder pathology and those articles reporting on other systems for upper-limb motion analysis.

## Conclusions

This study supports previous investigations that describe inertial sensors as a useful device to analyze upper-limbs kinematic. It identified movement patterns that show the relationship between the humerus and scapula in both abduction and flexion shoulder joint complex movements. Future studies with a bigger sample and subjects with pathological shoulders are need to determine differences in shoulder kinematics between healthy subjects and those suffering from shoulder pathology.
